# A Vehicular Mobile Standard Instrument for Field Verification of Traffic Speed Meters Based on Dual-Antenna Doppler Radar Sensor

**DOI:** 10.3390/s18041099

**Published:** 2018-04-05

**Authors:** Lei Du, Qiao Sun, Changqing Cai, Jie Bai, Zhe Fan, Yue Zhang

**Affiliations:** Division of Mechanics and Acoustics, National Institute of Metrology, Beijing 100029, China; sunq@nim.ac.cn (Q.S.); caichq@nim.ac.cn (C.C.); baijie@nim.ac.cn (J.B.); fanzhe@nim.ac.cn (Z.F.); zhangy@nim.ac.cn (Y.Z.)

**Keywords:** traffic speed enforcement, traffic speed meters, standard speed-measuring instrument, dual-antenna Doppler radar sensor, installation deviation correction, field verification

## Abstract

Traffic speed meters are important legal measuring instruments specially used for traffic speed enforcement and must be tested and verified in the field every year using a vehicular mobile standard speed-measuring instrument to ensure speed-measuring performances. The non-contact optical speed sensor and the GPS speed sensor are the two most common types of standard speed-measuring instruments. The non-contact optical speed sensor requires extremely high installation accuracy, and its speed-measuring error is nonlinear and uncorrectable. The speed-measuring accuracy of the GPS speed sensor is rapidly reduced if the amount of received satellites is insufficient enough, which often occurs in urban high-rise regions, tunnels, and mountainous regions. In this paper, a new standard speed-measuring instrument using a dual-antenna Doppler radar sensor is proposed based on a tradeoff between the installation accuracy requirement and the usage region limitation, which has no specified requirements for its mounting distance and no limitation on usage regions and can automatically compensate for the effect of an inclined installation angle on its speed-measuring accuracy. Theoretical model analysis, simulated speed measurement results, and field experimental results compared with a GPS speed sensor with high accuracy showed that the dual-antenna Doppler radar sensor is effective and reliable as a new standard speed-measuring instrument.

## 1. Introduction

Overspeed is a key factor in a high percentage of traffic accidents [1,2], and traffic speed surveillance is an important civilian application to improve road control, law enforcement, and the personal safety of drivers [3,4,5,6,7]. Traffic speed meters are important legal measuring instruments specially used for traffic speed surveillance and enforcement in China and around the world [8,9,10]. Until now, there have been four kinds of traffic speed meters legally used in China, i.e., across-the-road (AcTR) radar [7,8], above-the-road (AbTR) radar [9], dual-beam lidar, and inductive loops [10], which together with cameras make up overspeed automatic monitoring systems to measure and record a great deal of information of overspeed vehicles for image forensics automatically. Accordingly, many traditional traffic speed meters, such as hand-held radar and lidar guns (which are still widely used in the United States and many other countries of the world [11]), which can merely measure and display the speed value of a vehicle without any other information, cannot be used for traffic speed enforcement in China any more. Since traffic speed meters are specially used for important evidentiary purposes in traffic speed enforcement cases, the speed-measuring performances must be sufficient enough to assure the court’s confidence in using the measurement results of these traffic speed meters. According to the provisions of Chinese metrology law, traffic speed meters must be annually verified to examine and test speed-measuring performances by provincial- or municipal-level metrology institutes to ensure that speed measurement results are more accurate and reliable.

Annual verification for traditional traffic speed meters is mainly examined and tested in the laboratory using simulation methods [8,11]. However, most traffic speed meters working together with cameras are fixedly installed on the roadside [8], above the road [9,10], or under the road [10], and it is difficult, and even impossible, to disassemble and remove these traffic speed meters from the road to the laboratory for annual verification. Therefore, annual verification for these traffic speed meters must change to be examined and tested in the field by using a vehicular mobile standard speed-measuring instrument (abbreviated to “standard instrument” hereafter) mounted at a test vehicle [8]. The non-contact optical speed sensor and the GPS speed sensor are now the two most common types of standard instruments for measuring reference speed in China [12]. Most metrology institutes have chosen the non-contact optical speed sensor as the standard instrument for many years. However, the non-contact optical speed sensor requires extremely high installation accuracy, and its speed-measuring error is nonlinear and uncorrectable. Some metrology institutes have begun to replace the non-contact optical speed sensor with the GPS speed sensor as the standard instrument for its capability of rapid installation and excellent speed-measuring accuracy [12,13,14,15]. However, the speed-measuring accuracy of the GPS speed sensor is rapidly reduced when the amount of satellites received by the GPS speed sensor is insufficient enough [12].

A new standard instrument using a dual-antenna Doppler radar sensor (DDRS) is proposed in this paper. The DDRS combines the advantages of the non-contact optical speed sensor with those of the GPS speed sensor and avoids their disadvantages. This paper is organized as follows. Section 2 analyzes the problems of the two traditional standard instruments. Section 3 introduces the speed-measuring error of the single-antenna Doppler radar sensor (SDRS) caused by installation deviation and then describes the speed-measuring principle and a symmetrical structure form of the DDRS. Section 4 presents the calibration methods of the DDRS. Section 5 provides a principle prototype of the DDRS, Doppler shift calibration results, and some field experimental results compared with a GPS speed sensor with high accuracy to validate the new standard instrument. Section 6 concludes the paper.

## 2. Traditional Standard Instruments

The annual field verification method for traffic speed meters used in China can be found in [8], where it is shown that the accuracy of the reference speed of the test vehicle measured by the standard instrument is very important and determines the field verification results of the traffic speed meter under test. At present, the non-contact optical speed sensor and the GPS speed sensor are the two traditional standard instruments widely used by metrology institutes in China. In this section, the requirements for the standard instruments are firstly introduced, and the problems of the two traditional standard instruments are then respectively analyzed.

In the ideal case, the reference speed measured by the standard instrument should be infinitely close to the true speed value of the test vehicle. However, the deviation between the reference speed and the true speed is unavoidable in the actual case, which is caused by installation error, systematic error, field test conditions, and the environment, etc. In order to reduce the deviation, the standard instrument used by metrology institutes must be calibrated in the laboratory yearly to correct errors in the laboratory part of the deviation. After calibration, the maximum permissible error (MPE) of speed measurement of standard instruments must be within the range between ±0.5 km/h at a speed value less than 50 km/h and ±1.0% at a speed value greater than or equal to 50 km/h in the laboratory test conditions and environment.

**Figure 1:**
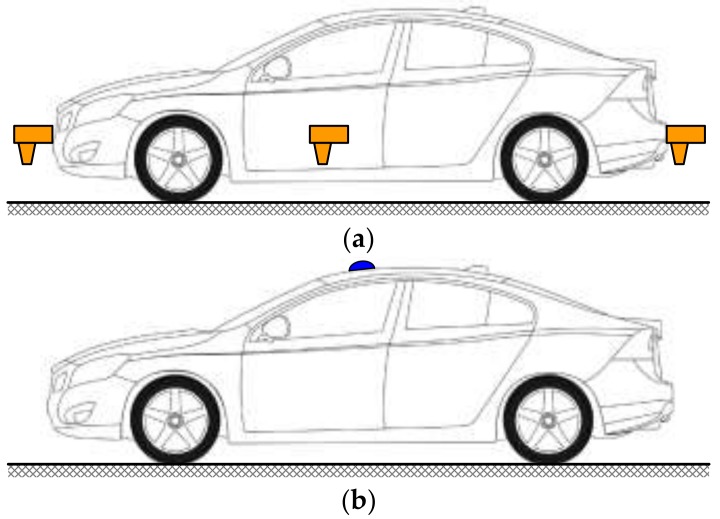
Schematic diagram of installation positions of standard instruments using various sensors: (**a**) the non-contact optical speed sensor; (**b**) the GPS speed sensor.

The non-contact optical speed sensor has been chosen as the standard instrument for about 20 years by most provincial- or municipal-level metrology institutes of China with the advantage of its mature technology, qualified speed-measuring accuracy and undoubted traceability. As shown in Figure 1a, the non-contact optical speed sensor can be mounted at the front, the rear, or the side of the test vehicle. However, there cannot be any occlusion between the sensor and the road surface, and the light emitted from the sensor must be perpendicular to the road surface. Furthermore, the mounting distance from the lower surface of the sensor to the road surface must be within the specified range. A higher or lower installation position and inclined installation angle will seriously reduce the speed-measuring accuracy of the standard instrument. Moreover, the speed-measuring error of the non-contact optical speed sensor is nonlinear and is not easily corrected by calibration methods in the laboratory. Therefore, the standard instrument using the non-contact optical speed sensor requires extremely high installation accuracy to ensure its speed-measuring accuracy, and a great deal of time and experience is needed to mount a non-contact optical speed sensor onto the test vehicle before field tests.

The GPS speed sensor has been chosen as the standard instrument for about 10 years by some metrology institutes of China with the advantages of rapid and easy installation, excellent speed-measuring accuracy, high sensitivity, and rapid response time [12,13,14,15]. However, the GPS speed sensor must receive signals from more than six GPS satellites to ensure its excellent speed-measuring accuracy [12]. Therefore, the sensor is usually mounted on the roof outside of the test vehicle as shown in Figure 1b and has no special requirements for its installation position and angle. However, the speed-measuring accuracy of the GPS speed sensor will be rapidly reduced when the amount of satellites received by the GPS speed sensor is insufficient [12]. Therefore, the standard instrument using the GPS speed sensor has many limitations on usage regions and cannot be used in indoor locations, urban high-rise regions, tunnels, mountainous regions, etc.

To avoid the above problems, a new standard instrument using the DDRS is proposed based on a tradeoff between the installation accuracy requirement and the usage region limitation, which combines the advantages of the non-contact optical speed sensor with those of the GPS speed sensor and avoids their disadvantages. The DDRS has no specified requirements for the mounting distance, little influence on its speed-measuring accuracy when installed on an inclined angle, and no limitation on the usage regions.

## 3. The Speed-Measuring Principle

In this section, the speed-measuring error of the SDRS caused by installation deviation is firstly introduced in brief. The speed-measuring principle of the DDRS is then described and a symmetrical structure form is proposed to simplify the calculation of speed measurement. Finally, the effect of installation deviation on the speed-measuring accuracy of the SDRS and the DDRS is analyzed by numerical comparisons.

### 3.1. The SDRS

**Figure 2:**
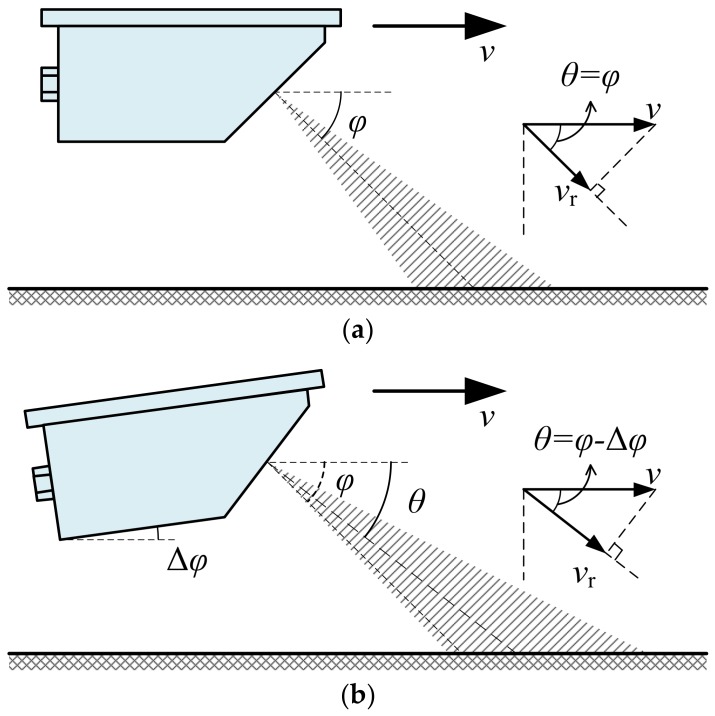
The simplified schematic diagram of the working principle of the SDRS: (**a**) the ideal situation without installation deviation; (**b**) the actual situation with installation deviation.

Doppler radar is widely used in traffic surveillance and safety control [16,17,18,19,20]. With the same installation position as that of the non-contact optical speed sensor, the Doppler radar sensor can also be mounted at the front, the rear, the side, or the bottom of the test vehicle as shown in Figure 1a. Theoretically, a single antenna is enough for the Doppler radar sensor to accurately measure the movement speed of the test vehicle based on the principle of the Doppler effect in the ideal situation without any installation deviation, as shown in Figure 2a. According to the Doppler shift formula of the SDRS [8,9], the movement speed value v can be expressed as follows:

(1) $$v=\frac{C\cdot f_{d}}{2f_{0}\cdot \cos\phi },$$

where $f_{d}$ is the Doppler shift, C is the speed of electromagnetic wave propagating in air, $f_{0}$ is the nominal emitted signal frequency of SDRS, and ϕ is called the nominal beam pointing angle (abbreviated to “nominal angle” hereafter) and defined as the included angle between the direction of movement speed and the nominal beam direction of the radar antenna as shown in Figure 2a.

However, installation deviation is obviously inevitable during the field tests in the actual situation as shown in Figure 2b. In such a situation, the actual beam pointing angle (abbreviated to “actual angle” hereafter) θ, which is defined as the included angle between the direction of movement speed and the actual beam direction of radar antenna as shown in Figure 2b, is no longer equal to ϕ, as is the ideal situation without installation deviation in Figure 2a, but varies with many factors, including installation deviation, bump, and pavement types, etc. Supposing there is an installation deviation angle Δϕ when the SDRS is mounted at the test vehicle, the actual angle θ equals ϕ−Δϕ in this situation as shown in Figure 2b. According to the Doppler shift formula of the SDRS [8,9], the movement speed value v can be expressed as follows:

(2) $$v=\frac{C\cdot f_{d}}{2f_{0}\cdot \cos\left(\phi -\Delta \phi \right)}.$$

Since it is impossible for the SDRS to measure and determine the installation deviation angle Δϕ in real time when it is tested in the field [8,9], the SDRS cannot measure the true value of movement speed v in actual situations.

One of the most widely used approximation method is to ignore the installation deviation angle Δϕ, which means using the fixed and known nominal angle ϕ instead of the variable and unknown actual angle θ to measure the movement speed of the test vehicle, so the measured value of movement speed $v_{m}$ can be approximately calculated with Equation (1) in the actual situation. Therefore, the relative measurement error $\delta_{v}$ of the SDRS based on the above approximation can be calculated as

(3) $$\delta_{v}=\frac{v_{m}-v}{v}=\frac{\cos\left(\phi -\Delta \phi \right)-\cos\phi }{\cos\phi }.$$

**Figure 3:**
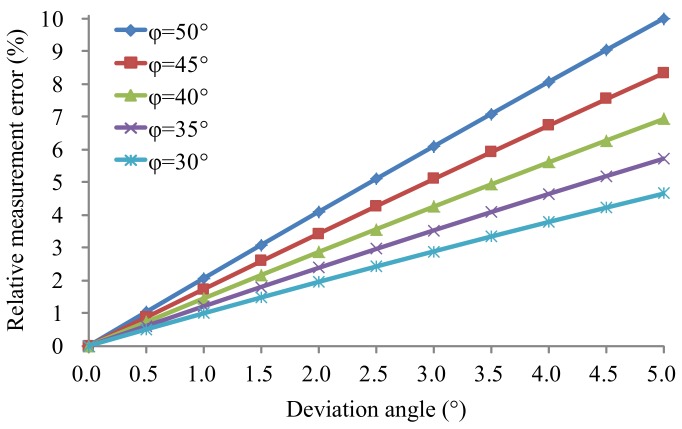
Relative measurement errors of the SDRS in the actual situation with different installation deviation angles.

Figure 3 shows the relative measurement errors of the SDRS theoretically calculated by Equation (3) with different installation deviation angles Δϕ and several typical nominal angles ϕ, where the nominal angle ϕ is chosen as 30°, 35°, 40°, 45°, or 50° and the deviation angles Δϕ range from 0 to 5°. It can be seen in Figure 3 that the relative measurement error increases rapidly with the increase of deviation angle and increases with the increase in nominal angle. To meet the requirement of a speed measurement MPE less than ±0.5 km/h (<50 km/h) or ±1.0% (≥50 km/h) for the standard instrument using the SDRS, the installation deviation angle must be smaller than 0.5° from Figure 3, which is an extremely high requirement for installation accuracy and even impossible to achieve during the field tests.

### 3.2. The DDRS

The speed measurement error of the SDRS is mainly due to the variable and unknown actual angle θ caused by installation deviation angle Δϕ in the actual situation. To reduce the speed measurement error and to compensate for the effect of installation deviation on speed-measuring accuracy, a standard instrument using a DDRS is proposed in this paper. In this subsection, the principle of speed measurement and installation deviation correction of the DDRS is firstly introduced, and a symmetrical structure form of the DDRS is then proposed to simplify the calculation of speed measurement.

**Figure 4:**
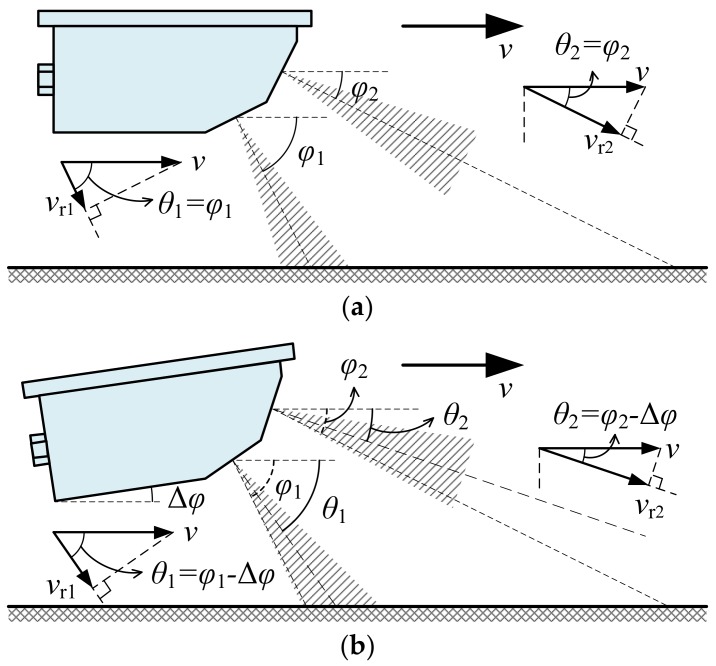
The simplified schematic diagram of the working principle of the DDRS: (**a**) the ideal situation without installation deviation; (**b**) the actual situation with installation deviation.

The simplified schematic diagram of the working principle of the DDRS is shown in Figure 4. The speed-measuring principle of the DDRS, as is that of the SDRS, is also based on the Doppler shift principle. The difference is that DDRS includes two separate antennas with nominal angles $\phi_{1}$ and $\phi_{2}$, respectively. When the DDRS mounted at the test vehicle moves at a speed value v along the road, its dual-antenna emits two individual single-frequency continuous-wave (SFCW) microwave signals with nominal emitted frequencies $f_{1}$ and $f_{2}$ to the road at the same time. According to the Doppler shift formula, two Doppler shifts $f_{d1}$ and $f_{d2}$ of the dual-antenna can be respectively expressed as

(4) $$\{\begin{array}{l} f_{d1}=\frac{2}{C}\cdot f_{1}\cdot v_{r1}\\ f_{d2}=\frac{2}{C}\cdot f_{2}\cdot v_{r2} \end{array},$$

where two radial speeds $v_{r1}$ and $v_{r2}$ are respectively equal to

(5) $$\{\begin{array}{l} v_{r1}=v\cdot \cos\theta_{1}\\ v_{r2}=v\cdot \cos\theta_{2} \end{array}.$$

After the correction of emitted frequencies and the calibration of Doppler shifts in the laboratory, which will be described in detail in the next section, the two radial speeds $v_{r1}$ and $v_{r2}$ can be respectively calculated as

(6) $$\{\begin{array}{l} v_{r1}=\frac{C}{2}\cdot \frac{f_{d1}}{f_{1}}\\ v_{r2}=\frac{C}{2}\cdot \frac{f_{d2}}{f_{2}} \end{array}.$$

As shown in Figure 4b, the actual angle $\theta_{1}$ equals $\phi_{1}-\Delta \phi$ and $\theta_{2}$ equals $\phi_{2}-\Delta \phi$ in the actual situation with an installation deviation angle Δϕ. According to Equation Sets (5) and (6), the true value of movement speed v can be written as

(7) $$\{\begin{array}{l} v=\frac{v_{r1}}{\cos\left(\phi_{1}-\Delta \phi \right)}\\ v=\frac{v_{r2}}{\cos\left(\phi_{2}-\Delta \phi \right)} \end{array}.$$

Theoretically speaking, the true value of movement speed v and installation deviation angle Δϕ can be accurately calculated by solving Equation Set (7). Therefore, the DDRS has the ability to compensate for the effect of installation deviation on speed-measuring accuracy and measure the true value of movement speed in the actual situation. However, the equations in Equation Set (7) are nonlinear and are related to trigonometric functions, and it is difficult to solve such equations to obtain exact solutions of v and Δϕ. To ensure real-time speed measurement, a symmetrical structure form of the DDRS is proposed to simplify the calculation of speed measurement, and an approximate solution of v is given based on the proposed structure form.

According to Equation Set (7), the relation between the true value of movement speed v and installation deviation angle Δϕ can be expressed as

(8) $$v=\frac{v_{r2}-v_{r1}}{2\sin\left(\frac{\phi_{1}+\phi_{2}}{2}-\Delta \phi \right)\cdot \sin\left(\frac{\phi_{1}-\phi_{2}}{2}\right)}.$$

**Figure 5:**
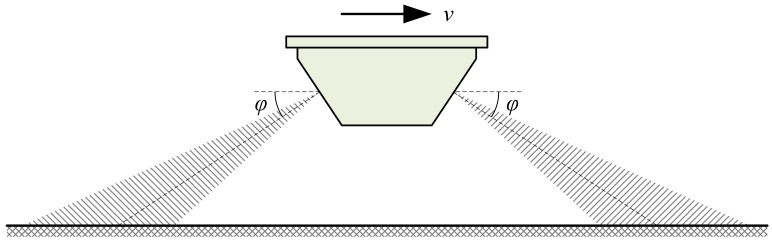
The simplified schematic diagram of the proposed symmetrical structure form of the DDRS.

If nominal angles $\phi_{1}$ and $\phi_{2}$ of the DDRS satisfy $\phi_{1}+\phi_{2}=\pi$, as shown in Figure 5, where $\phi_{1}=\phi$ and $\phi_{2}=\pi -\phi$, then Equation (8) can be simplified as

(9) $$v=\frac{v_{r1}-v_{r2}}{2\cos\Delta \phi \cdot \cos\phi }.$$

When the installation deviation angle Δϕ satisfies |Δϕ| ≤ 8°, then cosΔϕ≈1, and the true value of movement speed v shown in Equation (9) can be approximated as

(10) $$v_{m}=\frac{v_{r1}-v_{r2}}{2\cos\phi }.$$

Therefore, for the proposed symmetrical structure form of the DDRS as shown in Figure 5, the measured value of movement speed $v_{m}$ can be calculated as Equation (10) and Equation Set (5), and the relative measurement error $\delta_{v}$ can be calculated as

(11) $$\delta_{v}=\frac{v_{m}-v}{v}=\cos\Delta \phi -1.$$

Compared with the relative measurement error of the SDRS shown in Equation (3), the relative measurement error of the DDRS only relates to the installation deviation angle Δϕ and is always non-positive.

### 3.3. Numerical Comparison

**Figure 6:**
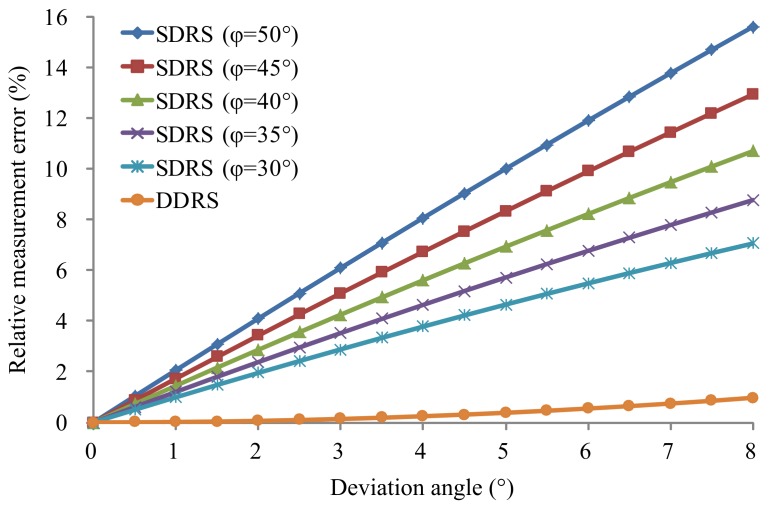
Absolute values of relative measurement errors of the SDRS and the DDRS in the actual situation with different installation deviation angles.

Figure 6 shows the absolute value comparison of relative measurement errors between the SDRS and the DDRS theoretically calculated Equations (3) and (11), with a nominal angle ϕ chosen as 30°, 35°, 40°, 45°, or 50°, and the deviation angles Δϕ range from 0 to 8°. The numerical comparison results are listed in Table 1.

Based on Figure 6 and Table 1, the relative measurement error of the DDRS is much smaller than that of the SDRS at the same deviation angle and increases much more slowly than that of the SDRS with the increase in deviation angle. Therefore, the DDRS can greatly reduce the speed measurement error compared with the SDRS and largely compensate for the effect of installation deviation on speed-measuring accuracy using the symmetrical structure form of the dual-antenna, as shown in Figure 5. To meet the requirement of a speed measurement MPE less than ±0.5 km/h (<50 km/h) or ±1.0% (≥50 km/h) for the standard instrument, the installation deviation angle of the DDRS must be smaller than 8° according to Table 1. Compared with the requirement of an installation deviation angle of the SDRS smaller than 0.5°, the DDRS has a requirement for installation accuracy that is easily achieved during field tests.

## 4. Calibration Methods

It can be deduced from Equation Set (6) that the accuracy of dual nominal emitted frequencies and dual Doppler shifts is very important to the calculation accuracy of radial speeds $v_{r1}$ and $v_{r2}$. Therefore, actual emitted frequencies must be measured to calibrate and correct the nominal emitted frequencies, and the dual Doppler shifts can also be simulated to calibrate the calculation accuracy of dual radial speeds of the DDRS in the laboratory before field tests.

### 4.1. Emitted Frequency Measurement

**Figure 7:**
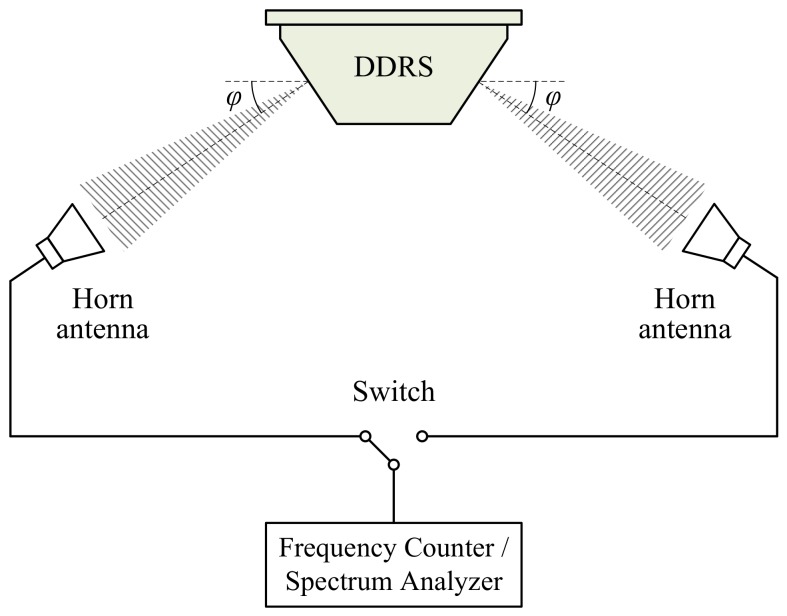
Block diagram of emitted frequency measurement system for the DDRS.

The block diagram of the emitted frequency measurement system designed for the DDRS is shown in Figure 7. The measurement system is composed of two horn antennas, a microwave frequency counter or spectrum analyzer, a microwave switch, and microwave cables. Two horn antennas are used to receive SFCW microwave signals from the dual-antenna of the DDRS. The microwave frequency counter or spectrum analyzer is used to measure the frequencies of the microwave signals received from the dual-antenna and is capable of measuring microwave frequencies ranging from 24,050 to 24,250 MHz with an uncertainty no greater than 1 part in 10^7^. The microwave switch is mainly used for the conversion of different horn antenna connections to the microwave frequency counter or spectrum analyzer. Microwave cables are mainly used for microwave signal transmission.

The measurement procedure can be found in [21,22]. Nominal emitted frequencies $f_{1}$ and $f_{2}$ in Equation Set (6) can be corrected by measurement results of actual emitted frequencies to ensure that the calculation results of radial speeds $v_{r1}$ and $v_{r2}$ are more accurate.

### 4.2. Doppler Shift Simulation

The dual Doppler shifts can be simulated to calibrate the calculation accuracy of dual radial speeds of the DDRS. However, both traditional tuning fork [11] and the single moving target simulator [8] are not suitable for calibrating the dual Doppler shifts of the DDRS. Therefore, a dual-antenna Doppler shift simulation system for the DDRS is specially designed to simulate and calibrate the symmetrical structure form of the DDRS as shown in Figure 5 in the laboratory.

**Figure 8:**
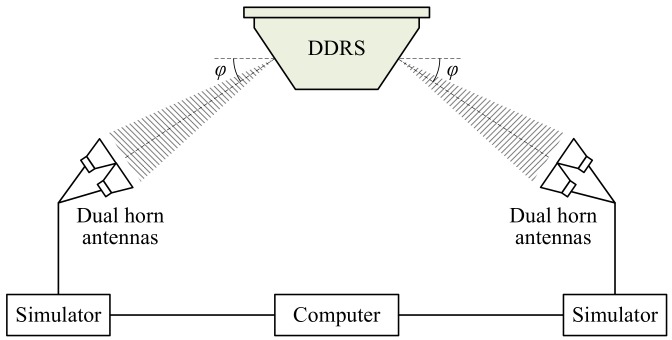
Block diagram of dual Doppler shift simulation system for the DDRS.

The block diagram of a dual-antenna Doppler shift simulation system for the DDRS is shown in Figure 8. The simulation system is composed of two groups of dual-horn antennas, two simulators, a computer, and cables. The two groups of dual-horn antennas are used to receive the SFCW microwave signals from the dual-antenna of the DDRS and emit the echo signals, including the Doppler shifts, back to it. The two simulators are used to simulate and generate two different Doppler shifts according to the simulated speed value and parameters of the DDRS. The computer is used to set and enter the intended simulated speed value and the parameters of the DDRS. The cables are mainly used for the connection between different parts of the simulation system.

**Figure 9:**
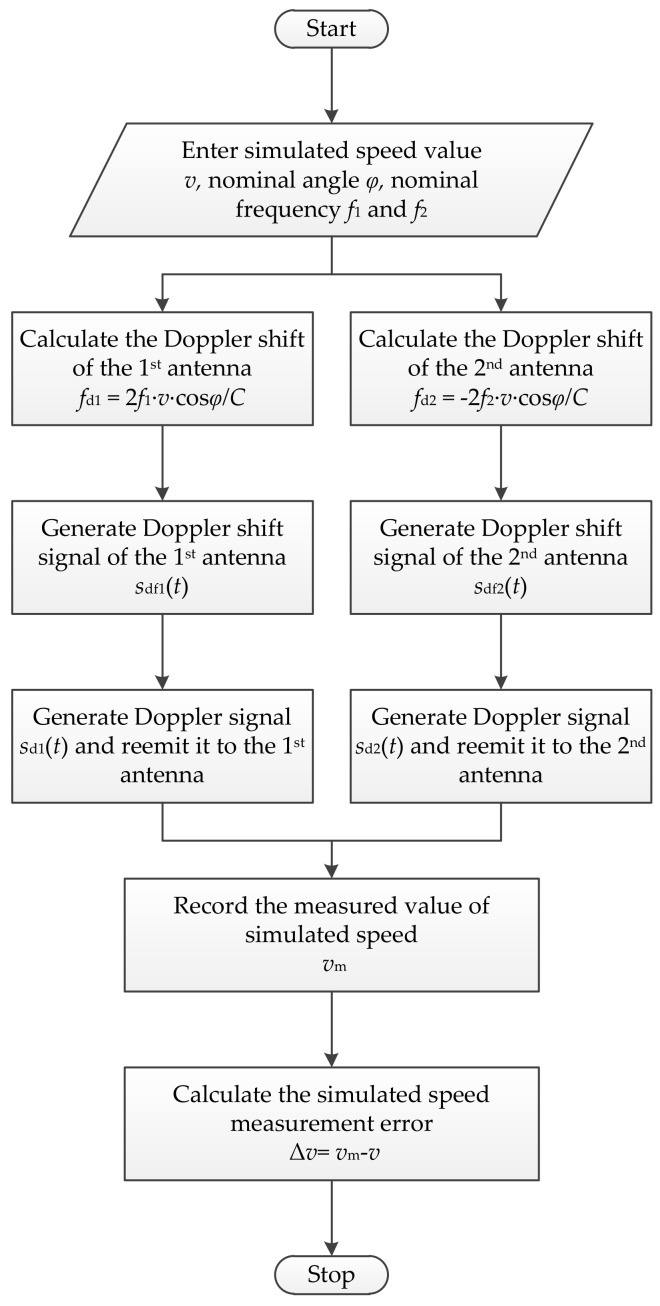
Flow diagram of dual Doppler shift simulation system for the DDRS.

The principle of the dual Doppler shift simulation system for the DDRS is based on the amplitude modulation (AM) of the emitted SFCW microwave signals from the dual-antenna of the DDRS. A flow diagram of the simulation system is given in Figure 9. The principle of the dual Doppler shift simulation is here analyzed and introduced in detail.

Firstly, the intended simulated speed value v and the parameters of the DDRS to be calibrated are entered into the computer, and the two ideal Doppler shifts $f_{d1}$ and $f_{d2}$ of the dual-antenna are then respectively calculated according to Equation Set (4) and given as

(12) $$\{\begin{array}{l} f_{d1}=\frac{2}{C}\cdot f_{1}\cdot v\cdot \cos\phi \\ f_{d2}=-\frac{2}{C}\cdot f_{2}\cdot v\cdot \cos\phi \end{array}.$$

Secondly, two Doppler shift signals $s_{\mathrm{df}1}\left(t\right)$ and $s_{\mathrm{df}2}\left(t\right)$ are respectively generated by the two simulators, which can be expressed as

(13) $$\{\begin{array}{l} s_{\mathrm{df}1}\left(t\right)=A_{\mathrm{df}1}\cdot \cos\left(2\pi f_{d1}t+\varphi_{\mathrm{df}1}\right)\\ s_{\mathrm{df}2}\left(t\right)=A_{\mathrm{df}2}\cdot \cos\left(2\pi f_{d2}t+\varphi_{\mathrm{df}2}\right) \end{array},$$

where $A_{\mathrm{df}1}$ and $A_{\mathrm{df}2}$ are the amplitudes of the two Doppler shift signals, and $\varphi_{\mathrm{df}1}$ and $\varphi_{\mathrm{df}2}$ are the phases.

Thirdly, the SFCW microwave signals $s_{1}\left(t\right)$ and $s_{2}\left(t\right)$ emitted from the dual-antenna of the DDRS are respectively received by the receiving antennas of the two simulators, which can be expressed as

(14) $$\{\begin{array}{l} s_{1}\left(t\right)=A_{1}\cdot \cos\left(2\pi f_{1}t+\varphi_{1}\right)\\ s_{2}\left(t\right)=A_{2}\cdot \cos\left(2\pi f_{2}t+\varphi_{2}\right) \end{array},$$

where $A_{1}$ and $A_{2}$ are the initial amplitudes of two emitted signals, and $\varphi_{1}$ and $\varphi_{2}$ are the initial phases, and then respectively amplitude-modulated by multiplying two Doppler shift signals $s_{\mathrm{df}1}\left(t\right)$ and $s_{\mathrm{df}2}\left(t\right)$. After filtering, two Doppler signals $s_{d1}\left(t\right)$ and $s_{d2}\left(t\right)$ are respectively generated by two simulators and can be expressed as

(15) $$\{\begin{array}{l} s_{d1}\left(t\right)=A_{d1}\cdot \cos\left[2\pi \left(f_{1}+f_{d1}\right)t+\varphi_{d1}\right]\\ s_{d2}\left(t\right)=A_{d2}\cdot \cos\left[2\pi \left(f_{2}+f_{d2}\right)t+\varphi_{d2}\right] \end{array},$$

where

(16) $$\{\begin{array}{l} A_{d1}=\frac{A_{1}\cdot A_{\mathrm{df}1}}{2}\\ A_{d2}=\frac{A_{2}\cdot A_{\mathrm{df}2}}{2} \end{array}$$

are the amplitudes of the two Doppler signals, and

(17) $$\{\begin{array}{l} \varphi_{d1}=\varphi_{1}+\varphi_{\mathrm{df}1}\\ \varphi_{d2}=\varphi_{2}+\varphi_{\mathrm{df}2} \end{array}$$

are the phases. It can be deduced from Equation Set (15) that the frequencies of the two Doppler signals are shifted to $f_{1}+f_{d1}$ and $f_{2}+f_{d2}$.

Finally, two Doppler signals are respectively reemitted to the dual-antenna of the DDRS by the emitting antennas of the two simulators, and the intended simulated speed is then measured by the DDRS. The measured value of simulated speed $v_{m}$ is recorded, and the simulated speed measurement error Δv is calculated and expressed as

(18) $$\Delta v=v_{m}-v.$$

By using the dual Doppler shift simulation system mentioned above, two Doppler shifts $f_{d1}$ and $f_{d2}$ in Equation Set (6) can be calibrated using simulated speed measurement results to improve the calculation accuracy of the dual radial speeds of the DDRS.

## 5. Realization and Experimental Results

To verify the speed-measuring principle and speed measurement error reduction of the DDRS, a principle prototype of the DDRS was specifically designed and realized for this study. In this section, the realization and main parameters of the principle prototype of the DDRS are described. A dual Doppler shift simulation system was designed for Doppler shift calibration of the DDRS in the laboratory, and the simulated speed measurement results of the principle prototype of the DDRS are given. Finally, for comparison, an experiment between a GPS speed sensor with high accuracy and the principle prototype of the DDRS was carried out in the field to evaluate the speed-measuring performances of the new standard instrument.

### 5.1. Principle Prototype of the DDRS

**Figure 10:**
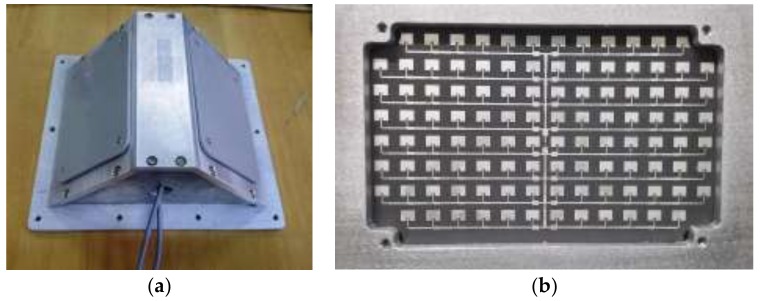
Photographs of the principle prototype of the DDRS: (**a**) the exterior; (**b**) the antenna.

**Figure 11:**
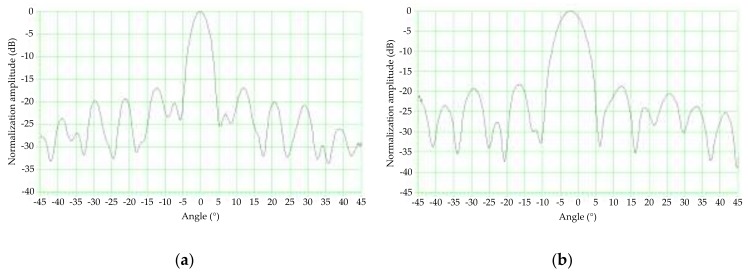
Antenna radiation patterns of the principle prototype: (**a**) vertical plane; (**b**) horizontal plane.

A photograph of the exterior of the principle prototype is shown in Figure 10a, and the antenna used in the principle prototype is shown in Figure 10b. The main parameters of the principle prototype are listed in Table 2. The principle prototype used the symmetrical structure form as shown in Figure 5, where $\phi_{1}$ = 45° and $\phi_{2}$ = 135°, and took microstrip array antennas instead of horn antennas as its dual antennas, with the same design parameters. To reduce the speed measurement error caused by the unknown triggering angle, the beamwidths were designed to be less than 6° and the sidelobe levels were designed to be lower than −15 dB in both horizontal and vertical planes [8]. One of the dual antennas was chosen to measure its antenna radiation patterns in the anechoic chamber at the traffic speed lab of the National Institute of Metrology, China. The antenna radiation pattern in the vertical plane is shown in Figure 11a, where the vertical beamwidth is 4.1° and the sidelobe level is −17.0 dB by calculation. The antenna radiation pattern in the horizontal plane is shown in Figure 11b, where horizontal beamwidth is 6.0° and sidelobe level is −18.2 dB by calculation.

To avoid mutual interference between the two individual microwave signals of the dual-antenna, the principle prototype used two different emitted frequencies, where $f_{1}$ = 24,150 MHz and $f_{2}$ = 24,125 MHz. The speed measurement range of the principle prototype was from 10 to 400 km/h, and the speed measurement MPE was ±0.25 km/h at a speed value less than 50 km/h and ±0.5% at a speed value greater than or equal to 50 km/h, which could satisfy the requirements of the speed measurement range and MPE as the standard instrument. The maximum refresh rate of the principle prototype was 20 Hz.

### 5.2. Doppler Shift Calibration Results

According to the design principle given in Section 4.2, a dual Doppler shift simulation system was designed for Doppler shift calibration of the DDRS in the laboratory. Main parameters of the simulation system are listed in Table 3. The simulation system included two target generators with the same design parameters and computer, where the target generator integrated transceiver horn antennas and simulator, as shown in Figure 8. Both of the target generators were designed to simulate the emitted frequency of the DDRS ranging from 24,050 to 24,250 MHz, and the beam pointing angle ranged from 0 to 180°, which could cover the design parameters of the principle prototype of the DDRS. The simulated speed range of the target generator was from 0 to 400 km/h, and the MPE of the simulated speed was ±0.01 km/h, which satisfied the requirements of the Doppler shift calibration of the DDRS in the laboratory.

**Figure 12:**
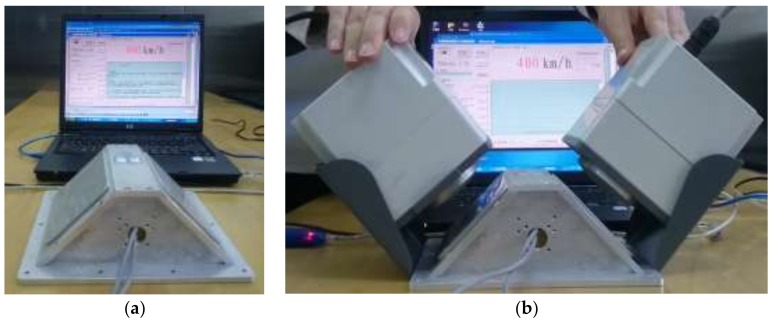
Photographs of the simulated speed measurement results. (**a**) At the initial value; (**b**) at 400.00 km/h.

Figure 12 shows the simulated speed measurement results at various intended speed points. For the first target generator, the simulated emitted frequency was set to 24,150 MHz, and the simulated beam pointing angle was set to 45°. For the second target generator, the simulated emitted frequency was set to 24,125 MHz, and the simulated beam pointing angle was set to 135°. It can be deduced from Figure 12a that the initial speed value of the principle prototype was 0 km/h, shown on the computer screen after the power was turned on. The intended simulated speed points were set to 10.00 km/h, 60.00 km/h, 100.00 km/h, 200.00 km/h, 300.00 km/h, and 400.00 km/h, respectively. As shown in Figure 12b, the transceiver horn antennas of the first target generator were placed close to the first antenna of the principle prototype of the DDRS while those of the second target generator were placed close to the second one. All simulated speed measurement errors were 0 km/h at the above simulated speed points, and Figure 12b shows the simulated speed measurement results at 400.00 km/h.

The numerical results of the simulated speed measurement at various intended speed points are given in Table 4 when the speed measurement resolution of the principle prototype was increased from 1 to 0.1 km/h. Five independent measurements were taken at every intended simulated speed point. The average of five speed-measuring values was taken as the measured value of the simulated speed, and the simulated speed measurement error was calculated. A calibration factor of 1.0001 was sent back to the Doppler shift calculation of the principle prototype to improve its radial speed-measuring accuracy based on the numerical results of the simulated speed measurements shown in Table 4.

### 5.3. Field Experimental Results

**Figure 13:**
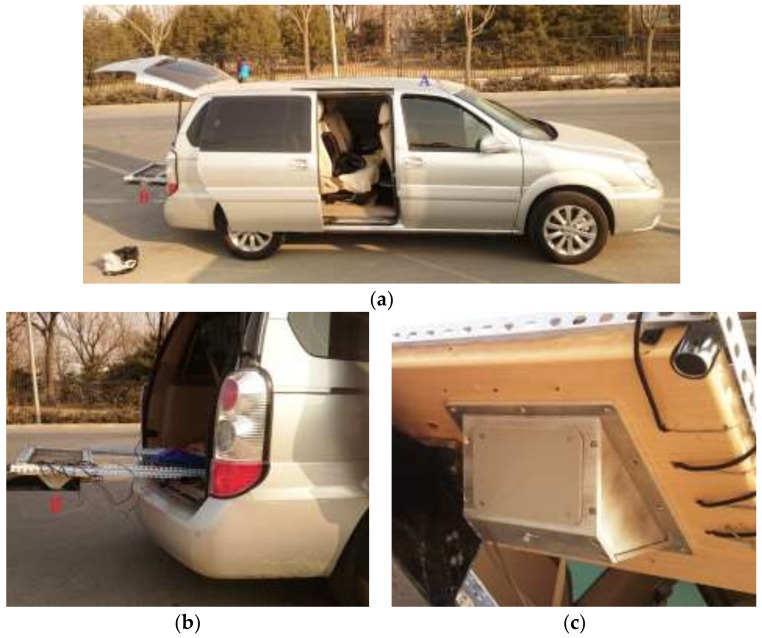
Field experiment. (**a**) Setup overview; (**b**) installation and fixation; (**c**) close-up of the principle prototype.

To evaluate the field speed-measuring performances of the proposed standard instrument using the DDRS, a comparison experiment between a GPS speed sensor with high accuracy and the principle prototype of the DDRS was carried out in the field. As shown in Figure 13, the two speed sensors were mounted at the same test vehicle, and measured the instantaneous movement speed of the test vehicle at the same time. The type of GPS speed sensor was Racelogic VBOX III (S/N 031183) with a refresh rate of 100 Hz. In accordance with the manufacturer’s instructions, the GPS antenna, which is marked A in Figure 13a, was mounted on the roof outside the test vehicle with an unrestricted view of the sky, and 8–10 satellites were available during the field testing process, which ensured that the speed measurement result of the GPS speed sensor was accurate and reliable. The principle prototype of the DDRS, which is marked B in Figure 13, was mounted at the bottom of a bracket at the rear of the test vehicle, as shown in Figure 13b, and no attention to height, angle, or level adjustment was paid during its installation process to evaluate the automatic compensation of the DDRS for the effect of installation deviation on the speed-measuring accuracy.

**Figure 14:**
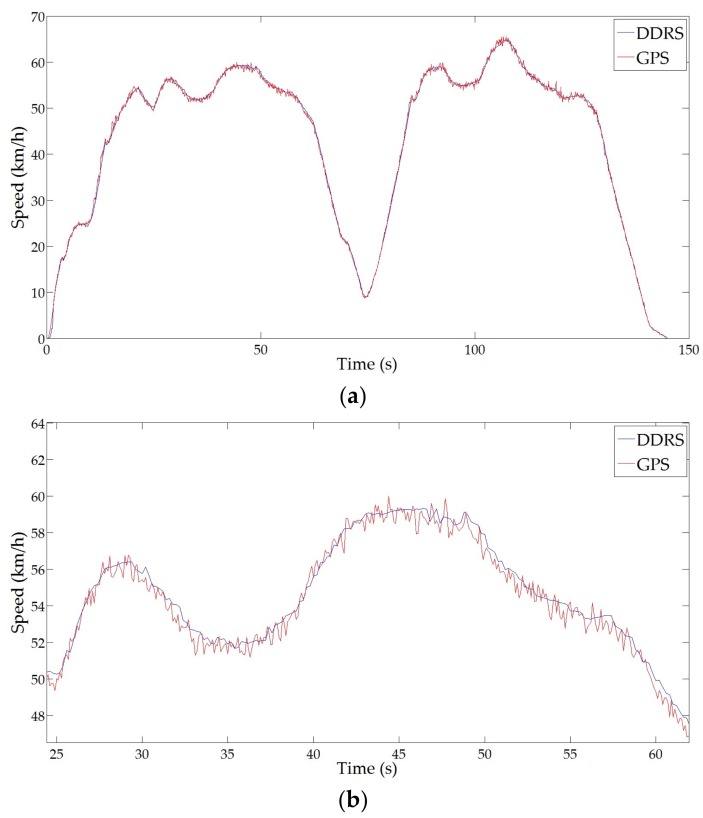

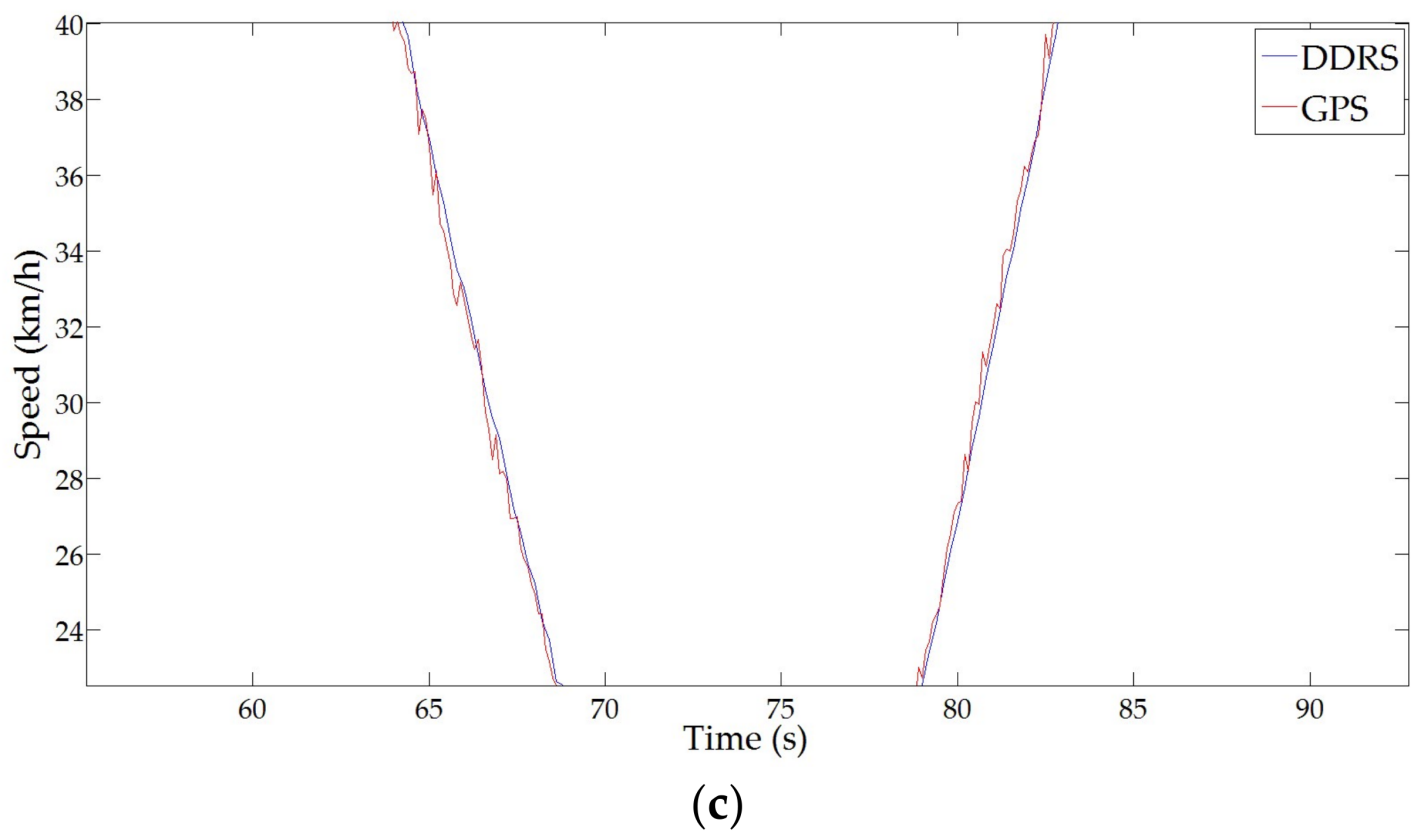
Experimental results. (**a**) Overview; (**b**) deviations in speed variation; (**c**) deceleration and acceleration.

According to the recommendation of the metrological field tests in OIML R 91 [23], a comparison experiment was carried out via an operating test in actual traffic with an overall study of possible errors due to complex factors affecting the measurement results, such as the shape of the antenna pattern, the distance change between the DDRS and the pavement, the reflection characteristics of different pavement types, braking or accelerating, vibration, and bumps caused by uneven pavement, etc. The test vehicle traveled on an asphalt road at the speed ranging from 0 to 65 km/h, with acceleration, deceleration, and approximate uniform motion during the field testing process. The duration of the comparison experiment was 145.0 s, and raw data of the experimental results are shown in Figure 14, where the red curve represents the speed measurement result of the GPS speed sensor and the blue curve represents that of the DDRS. Since more than six satellites were available during the field testing process, the speed-measuring values of the GPS speed sensor shown in Figure 14 were accurate and reliable [12]. Based on Figure 14a, there was a good overall correlation between the DDRS and the GPS speed sensor in general, showing that the DDRS, as a vehicular mobile standard instrument, effectively and reliably measured the reference speed of the test vehicle. However, based on Figure 14b, the speed measurement result of the GPS speed sensor was more meticulous and detailed in terms of describing speed variation compared with the DDRS result, and there were deviations between the speed-measuring values of the GPS speed sensor and those of the DDRS, especially amid high speed variation. This is mainly caused by the different refresh rates of the two speed sensors, where the refresh rate of the GPS speed sensor is 100 Hz, and that of the DDRS is 20 Hz. Since the test vehicle traveled in actual traffic, it was impossible for it to keep moving at a constant speed due to the complex actual traffic conditions, and the GPS speed sensor was more responsive to speed variation than the DDRS was because of the higher refresh rate of the GPS speed sensor.

A validation example is given in Figure 14c and Table 5. As shown in Figure 14c, the test vehicle decelerated from 59.0 to 69.0 s and accelerated from 75.0 to 85.0 s. The numerical comparison results of the speed-measuring values with respect to the GPS speed sensor and the DDRS during the above decelerations and accelerations are given in Table 5. It can be seen in Table 5 that the speed-measuring values of the DDRS were always higher than those of the GPS speed sensor during deceleration and were always lower than those of the GPS speed sensor during acceleration. When the test vehicle was decelerating, the GPS speed sensor responded more quickly to speed reduction than the DDRS did, and this explains why its speed-measuring values were always lower than those of the DDRS. The GPS speed sensors also responded more quickly when the test vehicle was accelerating. 

To evaluate the consistency of the speed-measuring values between the GPS speed sensor and the DDRS, the two speed measurement results were statistically investigated. The speed values measured by the GPS speed sensor and the DDRS at the same time in Figure 14a were chosen to calculate the speed-measuring deviations between these two speed sensors. The mean value of the deviations was 0.013 km/h, which indicated a good consistency in speed measurement between the GPS speed sensor and the DDRS. However, the standard deviation was 0.60 km/h, which indicated a high deviation range of speed measurement between the two sensors. This is partly caused by the different refresh rates of these two sensors. Another reason is that the speed-measuring value of the DDRS, because of the bumps and vibrations of bumpy roads, often jumped around the range of ±1 km/h.

## 6. Conclusions

In this paper, a new standard instrument is proposed for annual field verification of traffic speed meters based on a tradeoff between installation accuracy requirement and usage region limitations. The new standard instrument combines the advantages of the two traditional standard instruments using the non-contact optical speed sensor and the GPS speed sensor, and avoids their disadvantages. The DDRS used in the new standard instrument has no specified requirements for its mounting distance and no limitation on usage regions, and can automatically compensate for the effect of an inclined installation angle on its speed-measuring accuracy. Based on the speed-measuring principle and calibration methods of the DDRS, a principle prototype was designed and realized, with a speed ranging from 10 to 400 km/h and a speed measurement MPE ± 0.25 km/h (<50 km/h) or ±0.5% (≥50 km/h), and was calibrated in the laboratory by the proposed dual Doppler shift simulation system. In field experimental results, where the DDRS was compared with a GPS speed sensor with high accuracy, the standard instrument using the DDRS effectively and reliably measured the reference speed of the test vehicle.

## Figures and Tables

**Table 1:** Relative measurement errors of the SDRS and the DDRS in the actual situation with different installation deviation angles.

Δ*φ* (°)	SDRS (%)					DDRS (%)
	*φ* = 50°	*φ* = 45°	*φ* = 40°	*φ* = 35°	*φ* = 30°	
0	0.00	0.00	0.00	0.00	0.00	0.00
0.5	1.04	0.87	0.73	0.61	0.50	0.00
1	2.06	1.73	1.45	1.21	0.99	−0.02
2	4.10	3.43	2.87	2.38	1.95	−0.06
3	6.10	5.10	4.25	3.53	2.88	−0.14
4	8.07	6.73	5.61	4.64	3.78	−0.24
5	10.01	8.34	6.93	5.72	4.65	−0.38
6	11.91	9.91	8.22	6.77	5.49	−0.55
7	13.78	11.44	9.48	7.79	6.29	−0.75
8	15.61	12.94	10.70	8.77	7.06	−0.97

**Table 2:** Main parameters of the principle prototype of the DDRS.

Parameter	Notation	Value
Nominal emitted frequency of the first antenna	$f_{1}$	24,150 MHz
Nominal emitted frequency of the second antenna	$f_{2}$	24,125 MHz
Nominal angle of the first antenna	$\phi_{1}$	45°
Nominal angle of the second antenna	$\phi_{2}$	135°
Vertical beamwidth of the dual antennas	/	4.0°
Horizontal beamwidth of the dual antennas	/	6.0°
sidelobe levels of the dual antennas	/	<−15.0 dB
Maximum value of speed measurement	$v_{\max}$	400 km/h
Minimum value of speed measurement	$v_{\min}$	10 km/h
MPE of speed measurement	$\Delta v_{\mathrm{MPE}}$	±0.25 km/h (v < 50 km/h); ±0.5% (v ≥ 50 km/h)
Maximum refresh rate	/	20 Hz

**Table 3:** Main parameters of dual Doppler shift simulation system for the DDRS.

Parameter	Value
Maximum value of simulated emitted frequency	24,250 MHz
Minimum value of simulated emitted frequency	24,050 MHz
Maximum value of simulated beam pointing angle	180°
Minimum value of simulated beam pointing angle	0°
Maximum value of simulated speed	400 km/h
Minimum value of simulated speed	0 km/h
MPE of simulated speed	±0.01 km/h

**Table 4:** Numerical results of the simulated speed measurements.

Intended Simulated Speed (km/h)	Independent Measurement Results (km/h)					Average Value (km/h)	Error (km/h)
	1	2	3	4	5		
10.00	10.0	10.0	10.0	10.0	10.0	10.00	0.00
60.00	60.0	60.0	59.9	60.0	60.0	59.98	−0.02
100.00	99.9	100.0	100.0	100.0	100.0	99.98	−0.02
200.00	199.9	200.0	200.0	199.9	200.0	199.96	−0.04
300.00	300.0	299.9	300.0	299.9	300.0	299.96	−0.04
400.00	399.9	400.0	399.9	400.0	399.9	399.94	−0.06

**Table 5:** Numerical results of speed-measuring values of the GPS speed sensor and the DDRS during deceleration and acceleration.

Deceleration				Acceleration			
Time (s)	DDRS (km/h)	GPS (km/h)	Deviation (km/h)	Time (s)	DDRS (km/h)	GPS (km/h)	Deviation (km/h)
59.0	51.5	51.4	0.1	75.0	9.1	9.6	−0.5
60.0	49.9	49.3	0.6	76.0	11.3	11.4	−0.1
61.0	48.6	47.9	0.7	77.0	14.5	14.8	−0.3
62.0	47.3	46.8	0.5	78.0	18.3	18.5	−0.2
63.0	44.6	44.3	0.3	79.0	22.6	22.8	−0.2
64.0	40.9	39.8	1.1	80.0	26.9	27.3	−0.4
65.0	37.0	36.8	0.2	81.0	31.5	32.0	−0.5
66.0	33.0	32.7	0.3	82.0	35.9	36.1	−0.2
67.0	29.1	28.1	1.0	83.0	40.7	41.1	−0.4
68.0	25.2	24.9	0.3	84.0	45.7	46.7	−1.0
69.0	21.7	21.6	0.1	85.0	51.0	52.1	−1.1
